# Supplementary material for: Rethinking Alzheimer’s: novel miRNAs illuminate a disease beyond the brain
Source: Mol Psychiatry. 2026 Feb 13;31(7):3595–604. doi: 10.1038/s41380-026-03487-6 (PMC13269133; doi:10.1038/s41380-026-03487-6)
Supplement: Supplementary file 1 — Supplemetary Figure Legends [file 41380_2026_3487_MOESM1_ESM.docx]

**Title:** Rethinking Alzheimer’s: Novel miRNAs Illuminate a Disease Beyond the Brain.

Novotný JS^1,2†^, Čarná M^1†^, Dammer EB^3^, Mao Z^3^, Stokin GB^1,4^

^1^Institute of Molecular and Translational Medicine, Faculty of Medicine and Dentistry, Palacký University Olomouc, Olomouc, Czech Republic

^2^Department of Psychology and Abnormal Psychology, Faculty of Education, Palacký University Olomouc, Czech Republic

^3^Department of Pharmacology and Chemical Biology and Biochemistry, Emory University School of Medicine, Atlanta, USA

^4^Department of Neurology, Gloucestershire Royal Hospital, Gloucestershire Hospitals NHS Foundation Trust, Gloucester, UK

^†^These authors contributed equally to this work.

**Corresponding author:**

Gorazd B. Stokin, email: [gbstokin@alumni.ucsd.edu](mailto:gbstokin@alumni.ucsd.edu), Institute of Molecular and Translational Medicine, Faculty of Medicine and Dentistry, Palacký University Olomouc, Hněvotínská 1333/5, 779 00 Olomouc, Czech Republic

**Extended Data Figure legends**

**Extended Data Figure 1 - Systematic search of the database to identify circulating miRNAs for the meta-analyses.**

Flowchart of the selection process for the studies of plasma, serum and blood miRNAs changed in AD patients compared with healthy subjects to be included in the analysis.

**Extended Data Figure 2** **- Newcastle-Ottawa Scale-based analysis.**

Newcastle-Ottawa Scale-based analysis of the quality of included studies.

**Extended Data Figure 3 - Barplot showing the rate of vote agreement.**

Stability of the deregulation trend of 194 AD-related miRNAs across studies included in the meta-analysis.

**Extended Data Figure 4 - Qualitative compound trend plot for 194 miRNAs with significant compound P-value and significant pseudo-T-score.**

Colored barplot shows number of studies in which miRNAs were up- and down-regulated.

**Extended Data Figure 5 – Meta-analysis based biological pathways changed in AD.**

The top 5 most significantly enriched Reactome database forecast biological pathways based on the targets of significantly up- (pink circles) and down- (green circles) regulated miRNAs in AD patients compared with healthy subjects following meta-analysis.

**Extended Data Figure 6 – WmiRNACNA based biological pathway changes in AD.**

The top 3 most significantly enriched Reactome database forecast biological pathways based on the targets of significantly changes miRNAs in modules E, F and K.

**Extended Data Figure 7 – Key AD-related miRNAs.**

Venn diagram showing the intersection of the most significant AD-related miRNAs identified by Amanida-derived meta-analysis and the 3 most AD-related clusters containing miRNAs with similar behavior identified by weighted miRNAs co-expression meta-analysis.
