# Supplementary figures and images for: Rethinking Alzheimer’s: novel miRNAs illuminate a disease beyond the brain
Source: Mol Psychiatry. 2026 Feb 13;31(7):3595–604. doi: 10.1038/s41380-026-03487-6 (PMC13269133; doi:10.1038/s41380-026-03487-6)

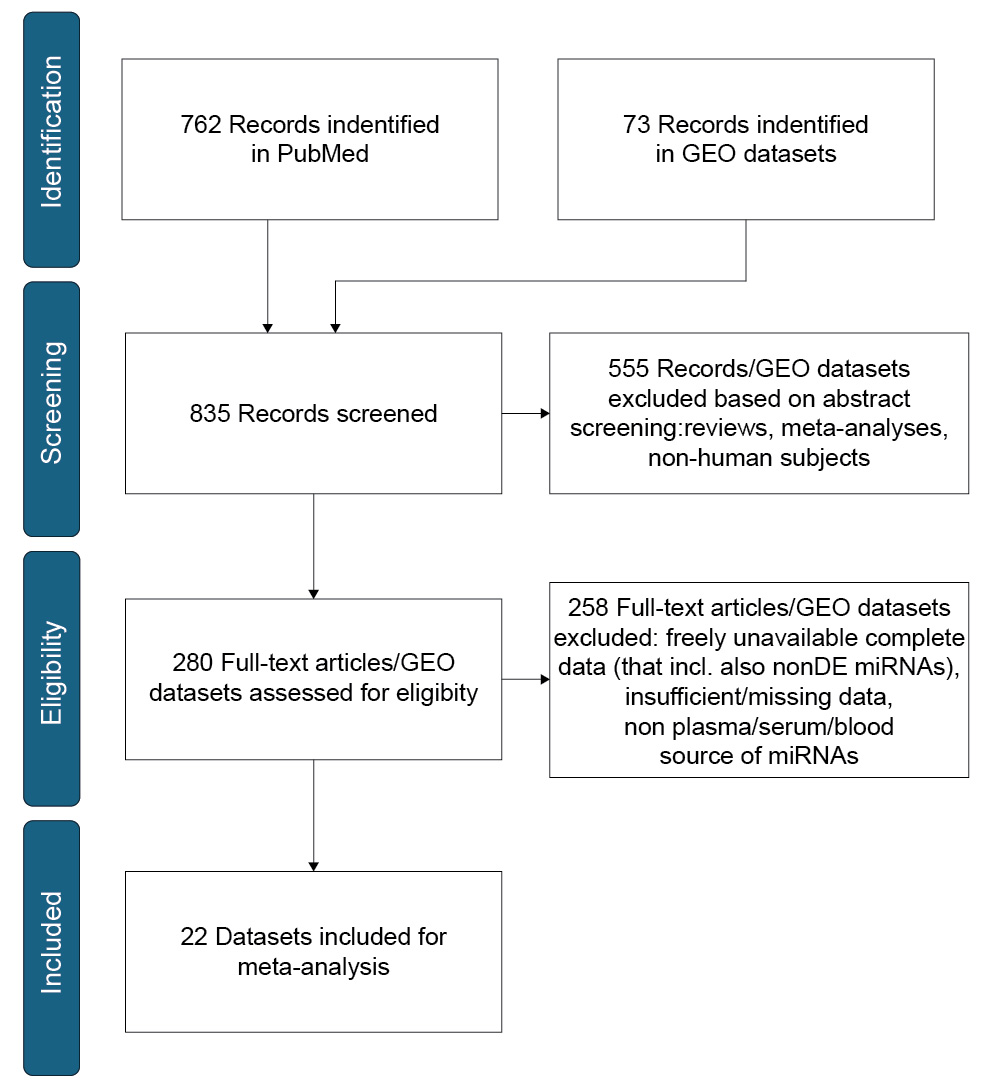

Supplement: Supplementary file 2 — Extended Data Figure 1 [file 41380_2026_3487_MOESM2_ESM.jpg]

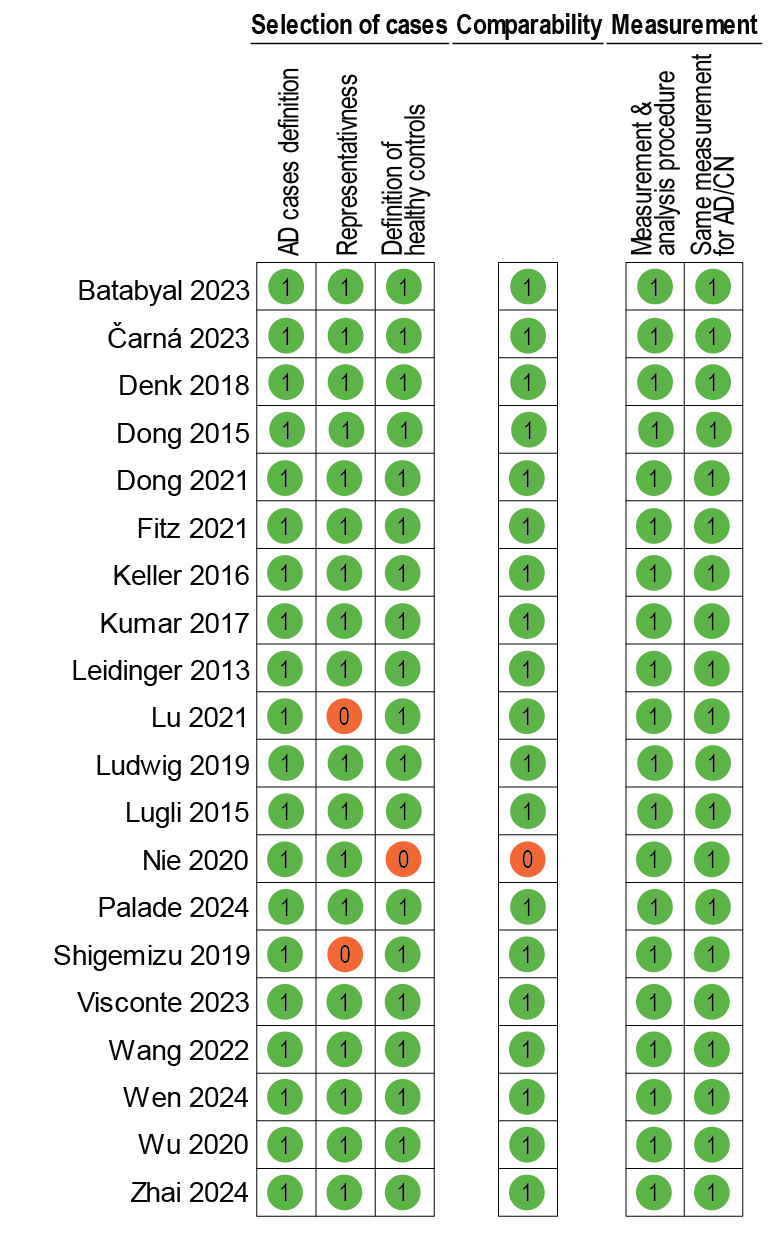

Supplement: Supplementary file 3 — Extended Data Figure 2 [file 41380_2026_3487_MOESM3_ESM.jpg]

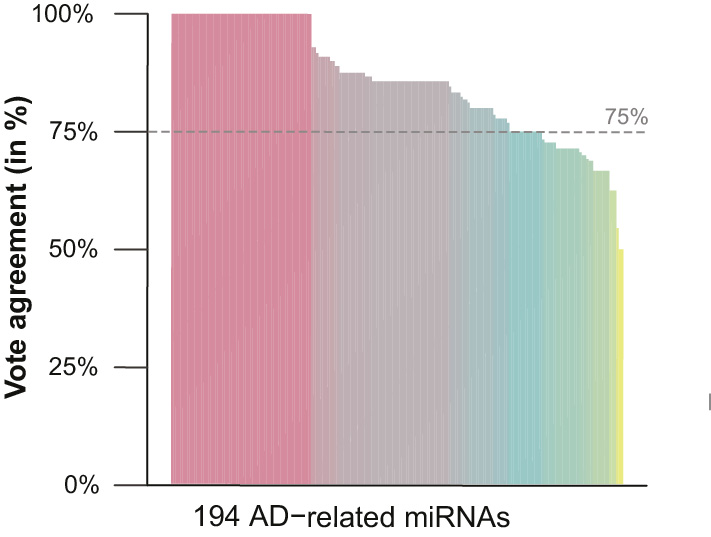

Supplement: Supplementary file 4 — Extended Data Figure 3 [file 41380_2026_3487_MOESM4_ESM.jpg]

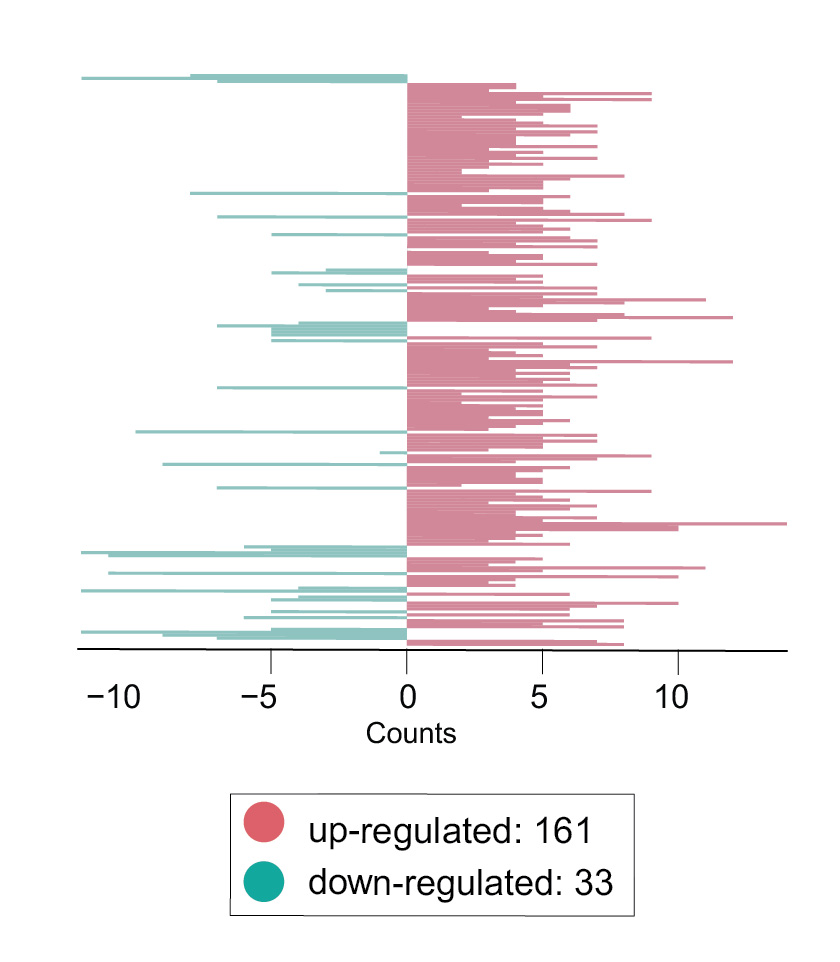

Supplement: Supplementary file 5 — Extended Data Figure 4 [file 41380_2026_3487_MOESM5_ESM.jpg]

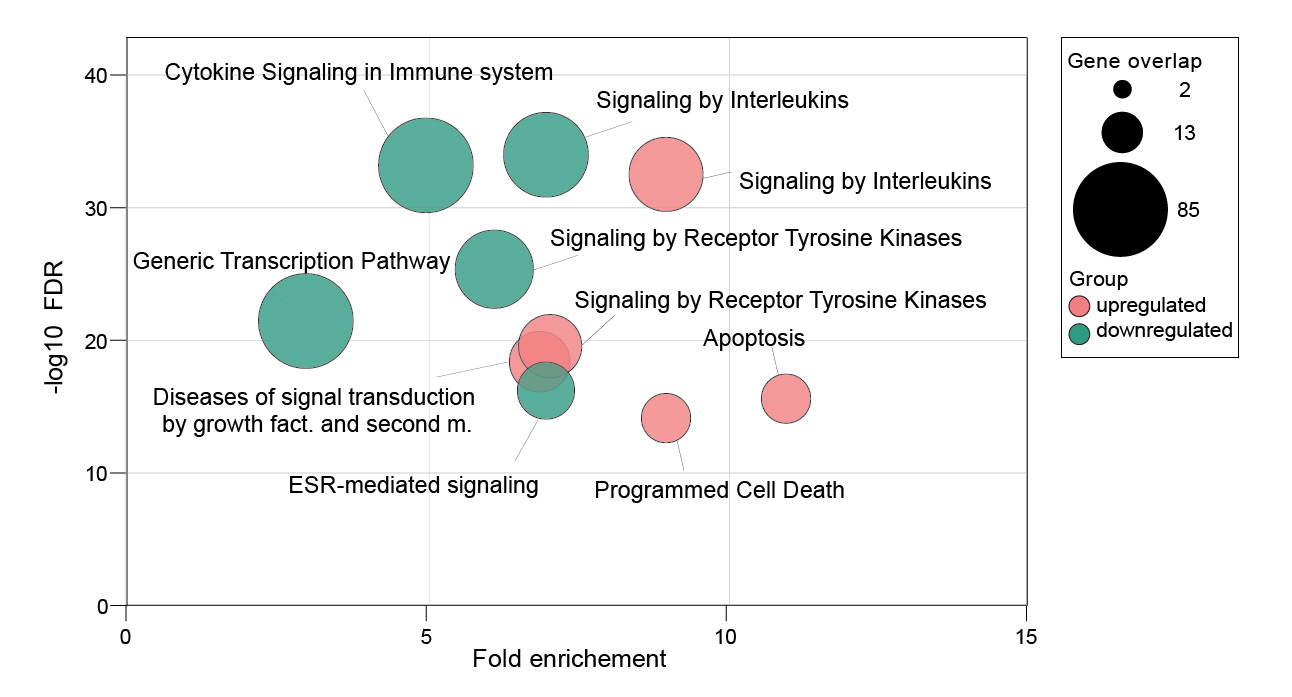

Supplement: Supplementary file 6 — Extended Data Figure 5 [file 41380_2026_3487_MOESM6_ESM.jpg]

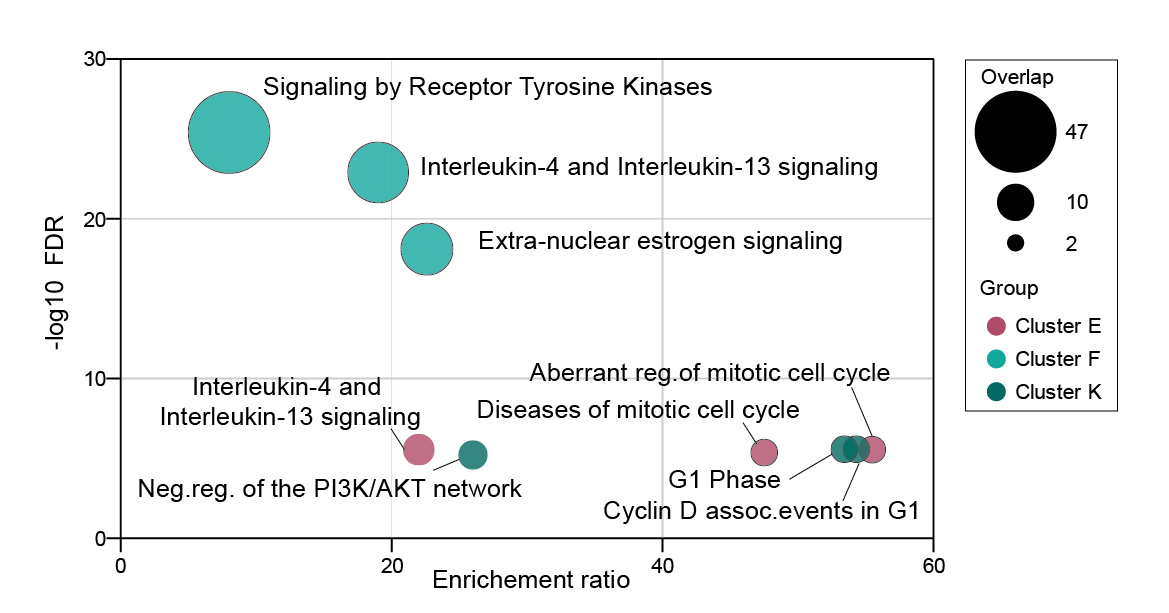

Supplement: Supplementary file 7 — Extended Data Figure 6 [file 41380_2026_3487_MOESM7_ESM.jpg]

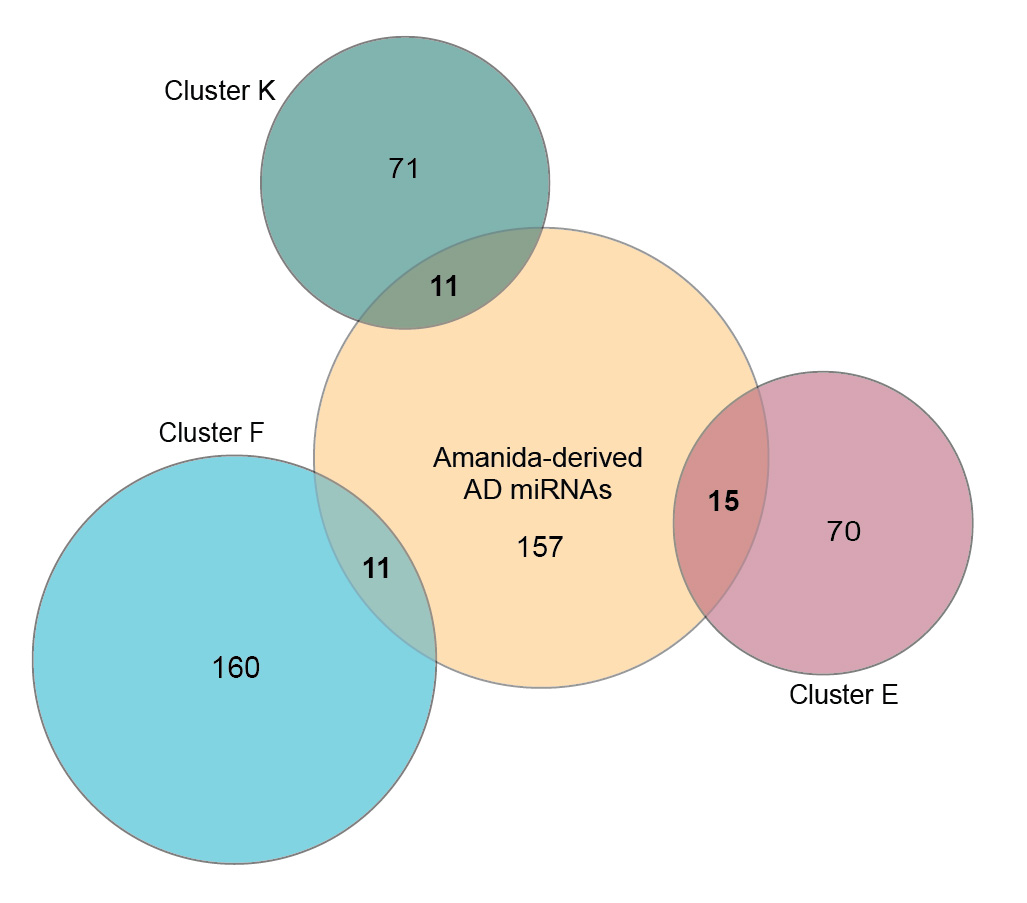

Supplement: Supplementary file 8 — Extended Data Figure 7 [file 41380_2026_3487_MOESM8_ESM.jpg]
